# Hypoglycemia in diabetes with low-affinity insulin antibody successfully improved by treating sleep apnea syndrome

**DOI:** 10.1210/jcemcr/luag077

**Published:** 2026-04-17

**Authors:** Kiyokazu Tanaka, Yujiro Nakano, Hisanori Goto, Miki Okumura, Yumie Takeshita, Toshinari Takamura

**Affiliations:** Department of Endocrinology and Metabolism, Kanazawa University Graduate School of Medical Sciences, Kanazawa, Ishikawa 920-8640, Japan; Department of Endocrinology and Metabolism, Kanazawa University Graduate School of Medical Sciences, Kanazawa, Ishikawa 920-8640, Japan; Department of Endocrinology and Metabolism, Kanazawa University Graduate School of Medical Sciences, Kanazawa, Ishikawa 920-8640, Japan; Department of Endocrinology and Metabolism, Kanazawa University Graduate School of Medical Sciences, Kanazawa, Ishikawa 920-8640, Japan; Department of Endocrinology and Metabolism, Kanazawa University Graduate School of Medical Sciences, Kanazawa, Ishikawa 920-8640, Japan; Department of Endocrinology and Metabolism, Kanazawa University Graduate School of Medical Sciences, Kanazawa, Ishikawa 920-8640, Japan

**Keywords:** hypoglycemia, insulin antibody, blood pH, acidosis

## Abstract

Glucose fluctuations, such as hypoglycemia, can cause atherosclerosis in patients with diabetes mellitus. Insulin antibodies with low affinity for insulin rarely cause blood glucose fluctuations, and no established treatment strategy currently exists. A 61-year-old man repeatedly experienced hyperglycemia in the afternoon and hypoglycemia from midnight to early morning. The patient had a history of insulin therapy for 20 years and received hemodialysis. Insulin antibodies with low affinity and high binding capacity were also detected. A positive correlation was observed between blood pH and fasting glucose levels. Sleep apnea syndrome remained untreated with continuous positive airway pressure owing to a deviated nasal septum, which reduced oxygen saturation while sleeping. Glycemic fluctuations improved after otolaryngologic surgery. Modeling of the insulin antibody in this patient suggested that reduced affinity resulted in the release of 8% of insulin from the antibody. Improvement in blood acidosis may help manage early morning hypoglycemia associated with insulin antibodies.

## Introduction

Diabetes mellitus silently accelerates atherosclerosis and causes severe complications, such as cardiovascular disease and cerebral infarction [1]. Several treatments can be used to reduce the risk of hyperglycemia. Overtreatment of diabetes causes hypoglycemia, which is life-threatening in the acute phase and damages arteries in the chronic phase [2].

Insulin antibodies are autoantibodies that cause diabetes mellitus. High-affinity insulin antibodies prevent insulin action and elevate blood glucose levels [3]. By contrast, low-affinity insulin antibodies cause fluctuations in blood glucose levels. Drugs, including inappropriate insulin injections and agents with thiol groups such as methimazole, may induce insulin antibody production. Genetic factors, including specific human leukocyte antigen genotypes [4, 5], also contribute to their development. Currently, no established treatment strategy exists for patients with insulin antibodies.

Here, we present the case of a patient who exhibited uncontrolled hyperglycemia and hypoglycemia with insulin antibodies. The blood pH-based approach successfully stabilized blood glucose levels. Analysis of the relationship between blood pH and glucose levels may provide clinical benefit in patients with insulin antibodies.

## Case presentation

A 61-year-old man was admitted to our hospital for recurrent hyperglycemia and hypoglycemia. He was diagnosed with diabetes mellitus at 40 years of age and was administered insulin analogs. Hypoglycemia was occasionally observed, and the insulin doses were titrated each time. Hemodialysis was administered at 60 years of age as he had developed diabetic nephropathy. Blood tests identified liver cirrhosis during the same period (Table 1). Continuous positive airway pressure (CPAP) was initiated for obstructive sleep apnea syndrome (oSAS) at that time.

**Table1:** Laboratory findings of the patient

Parameter	Values	Reference
WBC	3310/µL	3900–9800/µL
Hb	129 g/L (12.9 g/dL)	135–176 g/L (13.5–17.6 g/dL)
Plt	9.5 × 10^4^/µL	13.1–36.2 × 10^4^/µL
γGTP	24 IU/L	<70 IU/L
AST	9 IU/L	10–40 IU/L
ALT	11 IU/L	5–40 IU/L
LDH	263 IU/L	124–222 IU/L
TP	69 g/L (6.9 g/dL)	67–83 g/L (6.7–8.3 g/dL)
Alb	39 g/L (3.9 g/dL)	38–52 g/L (3.8–5.2 g/dL)
BUN	16 mmol/L (45 mg/dL)	2.8–7.8 mmol/L (8–22 mg/dL)
Cre	778.8 µmol/L (8.81 mg/dL)	53.9–91.9 µmol/L (0.61–1.04 mg/dL)
Na	137 mmol/L (137 mEq/L)	136–147 mmol/L (136–147 mEq/L)
K	4.0 mmol/L (4.0 mEq/L)	3.6–5.0 mmol/L (3.6–5.0 mEq/L)
Ca	2.07 mmol/L (8.3 mg/dL)	2.12–2.54 mmol/L (8.5–10.2 mg/dL)
IP	0.83 mmol/L (2.6 mg/dL)	0.77–1.38 mmol/L (2.4–4.3 mg/dL)
TC	1.68 mmol/L (65 mg/dL)	3.87–5.66 mmol/L (150–219 mg/dL)
HDL-C	0.98 mmol/L (38 mg/dL)	1.03–2.22 mmol/L (40–86 mg/dL)
TG	0.62 mmol/L (55 mg/dL)	0.56–1.68 mmol/L (50–149 mg/dL)
HbA1c	66 mmol/mol (8.2%)	26.7–44.2 mmol/mol (4.6–6.2%)
GA	(32.1%)	(12.4–16.3%)
Glucagon	26.6 ng/L (26.6 pg/mL)	5.4–55 ng/L (5.4–55 pg/mL)
Glucose	3.94 mmol/L (71 mg/dL)	
Insulin	4281.2 pmol/L (703 µU/mL)	
C-peptide	6.02 nmol/L (18.2 ng/mL)	
Glucose (morning)	4.55 mmol/L (82 mg/dL)	
Glucose (evening)	12.87 mmol/L (232 mg/dL)	
Insulin (morning)	3714.9 pmol/L (610 µU/mL)	
Insulin (evening)	3714.9 pmol/L (610 µU/mL)	

Abbreviations: Alb, albumin; ALT, alanine aminotransferase; AST, aspartate aminotransferase; BUN, blood urea nitrogen; Ca, calcium; Cre, creatinine; GA, glycoalbumin; Hb, hemoglobin; HbA1c, glycated hemoglobin; HDL-C, high-density lipoprotein cholesterol; IP, inorganic phosphorus; K, potassium; LDH, lactate dehydrogenase; Na, sodium; Plt, platelet; TC, total cholesterol; TG, triglyceride; TP, total protein; WBC, white blood cell; γGTP, gamma glutamyl transferase.

**Figure 1:**
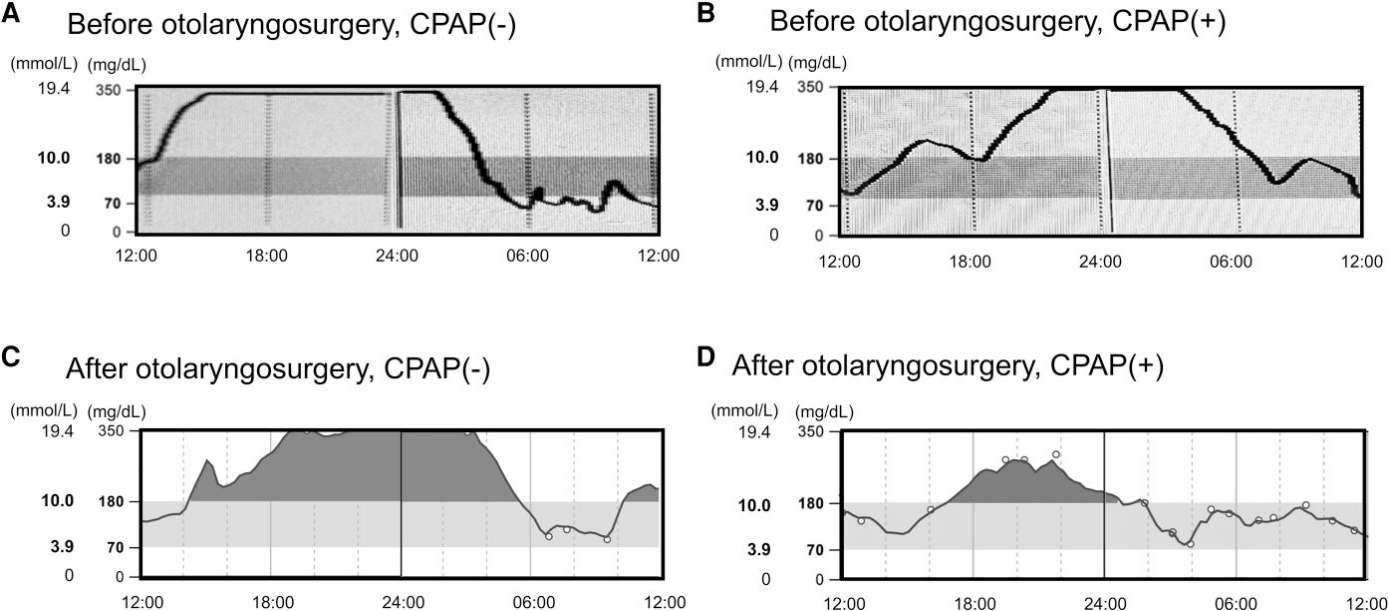
Continuous glucose monitoring before and after treatment. Representative glucose levels before treatment (A), during CPAP therapy (B), after otolaryngologic surgery without CPAP (C), and after otolaryngologic surgery with CPAP (D). The x-axis indicates time. The y-axis represents glucose levels. Glucose levels were measured using the flush glucose monitoring system (FreeStyle Libre^Ⓡ^, Abott). The average decline velocities of glucose at night were 200 mg/dL/h (SI: 11.1 mmol/L/h), 150 mg/dL/h (SI: 8.3 mmol/L/h), 150 mg/dL/h, and 80 mg/dL/h (SI: 4.4 mmol/L/h) in A, B, C, and D, respectively. Abbreviation: CPAP, continuous positive airway pressure.

Plasma glucose (PG) levels increased to >300 mg/dL (SI: 16.6 mmol/L) in the afternoon and decreased to <50 mg/dL (SI: 2.7 mmol/L) by midnight. Although all hypoglycemic agents, including insulin, were discontinued, the brittle glycemic fluctuations continued (Fig. 1). Blood tests showed glycated hemoglobin, fasting PG, insulin, and C-peptide levels of 8.2% (SI: 66.0 mmol/mol), 71 mg/dL (SI: 3.9 mmol/L), 703.0 µU/mL (SI: 4281.2 pmol/L), and 18.20 ng/mL (SI: 6.02 nmol/L), respectively (Table 1). Before breakfast, PG and insulin levels were 82 mg/dL (SI: 4.5 mmol/L) and 610 µU/mL (SI: 3714.9 pmol/L), respectively. Before dinner, PG and insulin were 232 mg/dL (SI: 12.8 nmol/L) and 610 µU/mL (SI: 3714.9 pmol/L), respectively (Table 1).

## Diagnostic assessment

**Figure 2:**
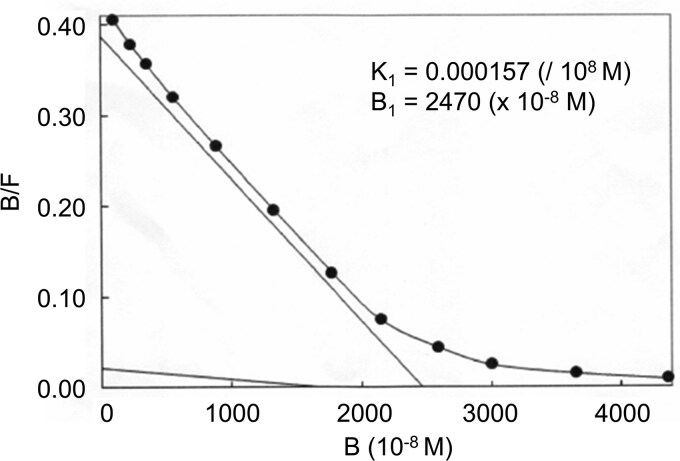
Scatchard plot of the insulin antibody. The x-axis represents the molar concentration of the bound complex. The y-axis shows the ratio of bound complex to free insulin. The value of K_1_ was calculated from the slopes of the plots. B_1_ represents the x-axis intercept calculated using linear superposition.

Insulin antibodies were detected in the patient's blood samples. The Scatchard plot analysis showed low affinity (K_1_= 0.000157/10^8^ M) and high binding capacity (B_1_= 2470 × 10^−8^ M) of the insulin antibody (Fig. 2). Compared with insulin antibodies in patients treated with regular insulin and patients with insulin autoimmune syndrome (affinity [/10^8^ M]: 3.14 ± 1.10 and 0.13 ± 0.05; binding capacity [×10^−8^ M]: 0.37 ± 0.18 and 23.7 ± 9.8, respectively) [6], he had lower affinity and higher binding capacity. Regardless of hemodialysis, brittle glucose fluctuations persisted. Acarbose, lixisenatide, diazoxide, and dexamethasone failed to stabilize daily glucose trends.

**Figure 3:**
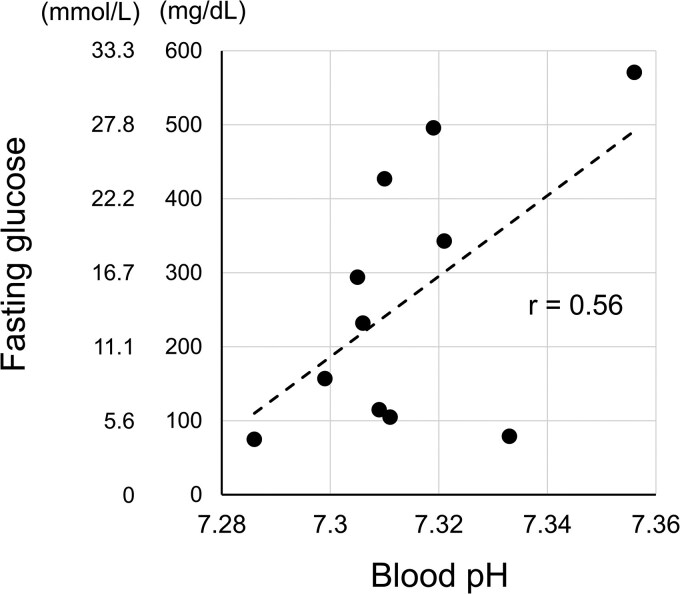
Relation between blood pH and fasting glucose level. Plasma glucose levels and blood pH were analyzed using blood samples collected randomly under fasting conditions. The correlation coefficient was calculated using Statistical Package for the Social Sciences software version 28.0 (IBM).

**Figure 4:**
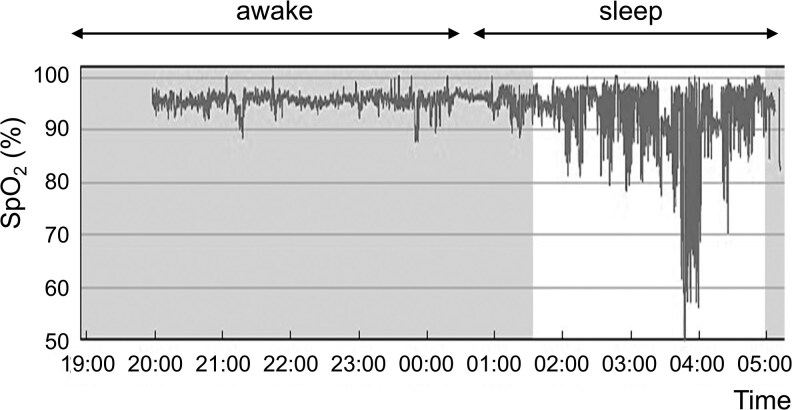
Polysomnography under CPAP. Night-time SpO_2_ levels were monitored after CPAP administration. The apnea–hypoxia index was 54.6. Abbreviations: CPAP, continuous positive airway pressure; SpO_2_, oxygen saturation.

Detailed observations revealed a positive correlation between blood pH and fasting glucose levels (Fig. 3). Polysomnography revealed low oxygen saturation during sleep because nasal congestion due to a deviated nasal septum compromised the efficacy of full facemask CPAP (SleepMate 10, Teijin, Tokyo, Japan) (Fig. 4). We hypothesized that the patient's oSAS accelerated blood acidosis, which caused insulin release from the antibodies at night.

**Figure 5:**
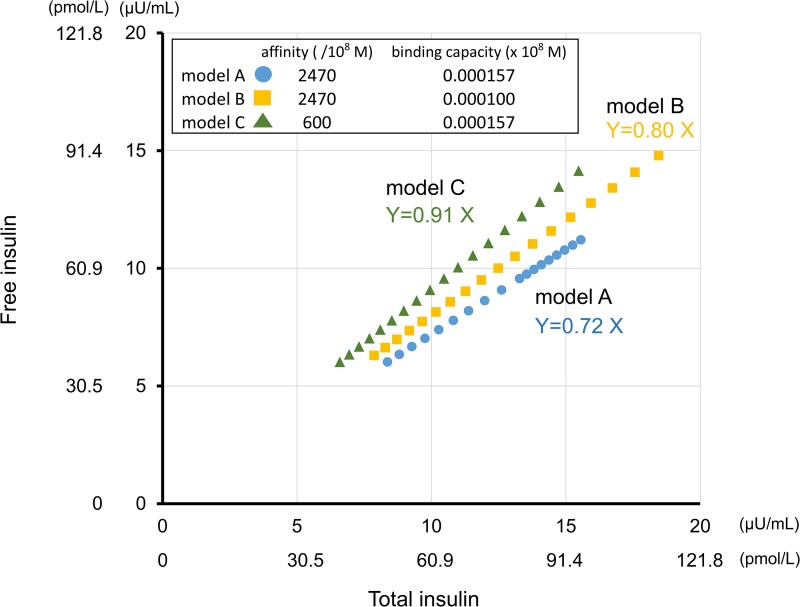
In silico modeling of predicting total and free insulin relation. The x-axis represents the total insulin concentration. The y-axis represents the free insulin concentration. Each plot was calculated using an equilibrium equation. In model A, 28% of the insulin was bound to the insulin antibody. In model B, 20% of the insulin was bound to the insulin antibody. In model C, 9% of the insulin was bound to the insulin antibody.

We simulated the free-to-total insulin ratio under different models of insulin antibodies, such as lower binding affinity or lower concentration than that of the patient's insulin antibody. The formula for the Scatchard plot was derived from the equilibrium equation describing insulin–antibody binding [7]. Accordingly, we calculated free insulin levels based on the total insulin level according to the antibody concentration and binding affinity specified in each model. The scatter plot in Fig. 5 was generated using the total and free insulin values calculated from three antibody models: model A, concentration 2470 × 10^−8^ M and binding affinity 0.000157/10^8^ M; model B, concentration 2470 × 10^−8^ M and binding affinity 0.000100/10^8^ M; and model C, concentration 600 × 10^8^ M and binding affinity 0.000157/10^8^ M. In model A, 28% of the insulin was bound to the antibody. In model B, 20% of insulin was bound to the antibody. In model C, 9% of the insulin was bound to the antibody. A shift from model A to model B resulted in the release of 8% of insulin from the antibody (Fig. 5).

## Treatment

**Figure 6:**
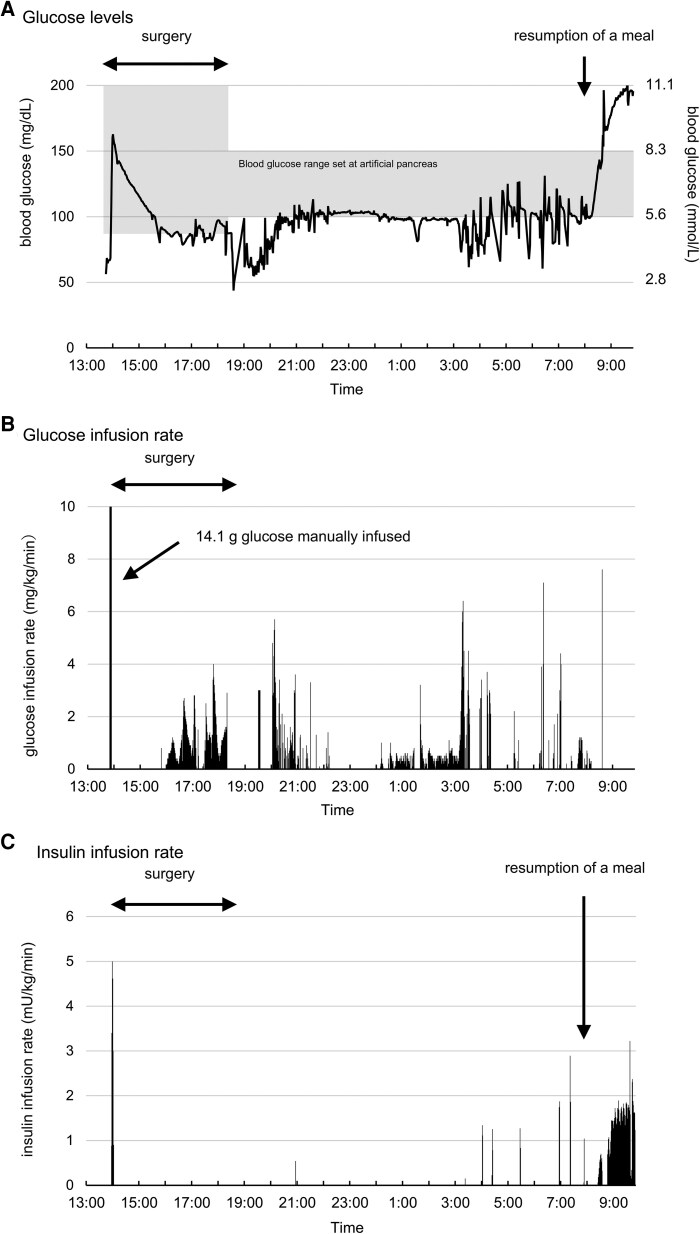
Glucose management with artificial pancreas in the perioperative phase. The artificial pancreas (model STG-55; Nikkiso) managed the glucose levels during surgery and overnight after surgery. Panels A, B, and C show glucose levels, glucose infusion rate, and insulin infusion rate, respectively. The gray shaded area in panel A indicates the target glucose range. A total of 14.1 g of glucose was manually infused immediately after starting the artificial pancreas. The total glucose infusion administered overnight was 15 g.

Otolaryngologic surgery was performed to correct a deflected nasal septum. During surgery, an artificial pancreas (model STG-55; Nikkiso, Tokyo, Japan) was used to titrate PG levels (Fig. 6). When PG levels fell below the lower limit or rose above the upper limit, 10% glucose or regular insulin was administered continuously until PG levels restored within the target range. The target PG level was set at 80-200 mg/dL (SI: 4.4-11.1 mmol/L) during surgery. After a manual bolus injection of glucose, glucose was intermittently administered, whereas insulin was withheld. The artificial pancreas continued to function overnight after the operation, with target PG levels ranging from 100 to 150 mg/dL (SI: 5.5-8.3 mmol/L). The total amount of glucose administered at night was 15 g. Insulin was administered after breakfast on the first postoperative day (Fig. 6).

## Outcome and follow-up

After otolaryngologic surgery, his glucose fluctuations flattened compared with preoperative patterns (Fig. 1).

## Discussion

Our patient had extremely high insulin levels. Insulin is usually cleared from the liver and the kidneys [8]. Insulin antibodies impair insulin clearance and promote insulin accumulation in the peripheral blood [9]. In the present case, the patient failed to clear insulin because of liver cirrhosis and end-stage renal dysfunction with a high titer of insulin antibodies. We hypothesized that the insulin antibody would not interfere with the assay of serum insulin levels, although we could not determine the contact site between insulin and the insulin antibody. An insulin assay was used to determine the levels of both free and antibody-bound insulin. Insulin levels remained extremely high under both hyperglycemic and hypoglycemic conditions. These results suggest that the insulin antibody in this patient captured a high insulin titer.

The patient experienced recurrent early-morning hypoglycemic episodes, suggesting nocturnal release of insulin from insulin antibodies. The equilibrium affinity decreases under acidic conditions [10]. Patients with SAS exhibit respiratory acidosis due to CO_2_ accumulation [11]. Accordingly, acidosis induced by SAS alters the affinity between insulin and insulin antibodies, increasing the amount of released insulin. Thus, uncontrolled SAS may cause insulin-dependent hypoglycemia in the early morning. Our observations clearly show a correlation between low blood pH and low PG levels. A previous case report showed that acidosis causes hypoglycemia in a patient with anti-insulin antibodies [12]. We estimated the amount of insulin released from antibodies based on changes in affinity. Calculations suggest that approximately 8% of antibody-bound insulin was released when affinity decreased.

Managing unstable glucose levels due to insulin antibodies is challenging. Previous studies suggested the use of several approaches. The first approach is preventing hypoglycemia by providing a glucose supply. Late-evening cornstarch may elevate blood glucose levels early in the morning [13]. Glucocorticoids promote hepatic gluconeogenesis and may prevent hypoglycemia [3]. The second approach is to reduce the secretion of endogenous insulin, which can reduce insulin capture and hypoglycemia caused by the released insulin. Alpha-glucosidase inhibitors and short-acting glucagon-like peptide 1 (GLP-1) analogs decrease postprandial glucose and insulin elevation [14]. The third approach is to reduce insulin antibody levels. Glucocorticoids may reduce autoantibody production [15]. Plasma exchange directly reduces autoantibodies [16]. A case report suggested that rituximab, an anti-cluster of differentiation 20 antibody, may reduce insulin antibody production [17]. In our case, an extremely high level of free insulin in the morning quickly decreased blood glucose levels and prevented hepatic gluconeogenesis, thereby negating the effects of cornstarch and dexamethasone. Alpha-glucosidase inhibitors and short-acting GLP-1 analogs failed to decrease hypoglycemic episodes because extremely high levels of insulin antibodies had already bound sufficient insulin to promote hypoglycemia. A previous case report suggested that correction of blood pH stabilizes the affinity of insulin antibodies and prevents sudden release of insulin [12]. Sodium bicarbonate administration successfully maintained blood glucose levels within 100-150 mg/dL (SI: 5.5-8.3 mmol/L). In this case report, several findings suggest that blood pH decreased at midnight because of SAS. Targeted treatment of SAS improved reduction in glucose levels at night. Assessment and treatment of acidosis may be recommended in patients with insulin antibodies.

In conclusion, we present the case of a patient who exhibited uncontrolled hyperglycemia and hypoglycemia with insulin antibodies. The blood-pH–based approach successfully stabilized blood glucose levels. Analysis of the relationship between blood pH and glucose levels is potentially helpful for patients with insulin antibodies.

## Learning points

- Unexpected hyperglycemia and hypoglycemia cycles suggest the presence of insulin antibodies with low binding affinity.
- Acidosis caused by sleep apnea syndrome may change binding affinity and lead to insulin release from the antibody.
- Stabilizing blood pH may be beneficial for fluctuations in circulating free insulin and glucose levels.

## Data Availability

Some or all datasets generated during and/or analyzed during the current study are not publicly available but are available from the corresponding author on reasonable request.

## Abbreviations

Alb: albumin
ALT: alanine aminotransferase
AST: aspartate aminotransferase
BUN: blood urea nitrogen
Ca: calcium
CPAP: continuous positive airway pressure
Cre: creatinine
GA: glycoalbumin
GLP-1: glucagon-like peptide 1
Hb: hemoglobin
HbA1c: glycated hemoglobin
HDL-C: high-density lipoprotein cholesterol
IP: inorganic phosphorus
K: potassium
LDH: lactate dehydrogenase
Na: sodium
oSAS: obstructive sleep apnea syndrome
PG: plasma glucose
Plt: platelet
SpO_2_: oxygen saturation
TC: total cholesterol
TG: triglyceride
TP: total protein
WBC: white blood cell
γGTP: gamma glutamyl transferase
